# Xylobiose Prevents High-Fat Diet Induced Mice Obesity by Suppressing Mesenteric Fat Deposition and Metabolic Dysregulation

**DOI:** 10.3390/molecules23030705

**Published:** 2018-03-20

**Authors:** Soo-min Lim, Eunju Kim, Jae-Ho Shin, Pu Reum Seok, Sangwon Jung, Sang-Ho Yoo, Yuri Kim

**Affiliations:** 1 Department of Nutritional Science and Food Management, Ewha Womans University, Seoul 03760, Korea; arlenalim@naver.com (S.-m.L.); eunju831@naver.com (E.K.); 2 Department of Biomedical Laboratory Science, Eulji University, Seongnam-si, Gyunggi-do 13135, Korea; shinjh@eulji.ac.kr (J.-H.S.); purumi523@naver.com (P.R.S.); 3 R&D Center, TS Corporation, Incheon 22300, Korea; chemjsw@ts.co.kr; 4 Department of Food Science and Biotechnology, and Carbohydrate Bioproduct Research Center, Sejong University, 209 Neungdong-ro, Gwangjin-gu, Seoul 05006, Korea; shyoo@sejong.ac.kr

**Keywords:** xylobiose, obesity, adipogenesis, lipogenesis, inflammation, mesenteric adipose tissue

## Abstract

Obesity is a public concern and is responsible for various metabolic diseases. Xylobiose (XB), an alternative sweetener, is a major component of xylo-oligosaccharide. The purpose of this study was to investigate the effects of XB on obesity and its associated metabolic changes in related organs. For these studies, mice received a 60% high-fat diet supplemented with 15% d-xylose, 10% XB, or 15% XB as part of the total sucrose content of the diet for ten weeks. Body weight, fat and liver weights, fasting blood glucose, and blood lipids levels were significantly reduced with XB supplementation. Levels of leptin and adipokine were also improved and lipogenic and adipogenic genes in mesenteric fat and liver were down-regulated with XB supplementation. Furthermore, pro-inflammatory cytokines, fatty acid uptake, lipolysis, and β-oxidation-related gene expression levels in mesenteric fat were down-regulated with XB supplementation. Thus, XB exhibited therapeutic potential for treating obesity which involved suppression of fat deposition and obesity-related metabolic disorders.

## 1. Introduction

Since 2004, the worldwide overweight population has increased approximately 20%, with a 1~2% increase observed with each decade [1]. In 2014, the World Health Organization (WHO, Geneva, Switzerland) reported that approximately 38% of the adult population worldwide is overweight with a body mass index (BMI) ≥ 25, and 11~15% of the adult population worldwide is obese, with a BMI ≥ 30. Obesity is a metabolic disease that is characterized by hypertrophy and hyperplasia of white adipose tissues (WAT) and is associated with chronic inflammation of fat and nonalcoholic fatty liver disease (NAFLD) [2,3,4]. Obesity can cause hormonal and immune dysfunctions, dyslipidemia, cardiovascular diseases, and a higher risk of insulin resistance that can be a risk factor for type 2 diabetes (T2DM) [5,6,7].

In an obese state, both fat synthesis (lipogenesis) and differentiation of pre-adipocytes into mature adipocytes (adipogenesis) are stimulated in liver and adipose tissues. Excessive lipogenesis is responsible for increases in adipocyte size due to triglyceride (TG) accumulation from fatty acids taken up from the circulation or by de novo synthesis [8,9]. With high levels of lipids in plasma, free fatty acids (FFAs) are not efficiently taken up by adipocytes for storage and this leads to hepatic accumulation of TGs and increased circulating levels of very low-density lipoprotein cholesterol (VLDL-C) [8,10,11].

Adipose tissue is an endocrine organ that produces and releases compounds and enzymes, including lipoprotein lipase (LPL), resistin, cholesteryl-ester transfer protein, adiponectin, and leptin [12,13]. Moreover, WAT is a major source of inflammatory cytokines and predominantly initiates inflammatory responses [5,14]. There are several types of adipose tissues, including subcutaneous, perirenal, mesenteric, and brown adipose tissues. Among these, mesenteric fat, a part of visceral fat, is called portal adipose tissue since pro-inflammatory cytokines and FFAs are released by it and they are directly drained to the portal vein. As a result of this drainage, mild liver steatosis, insulin resistance, and systemic inflammation can occur [15,16,17]. It has been demonstrated that mesenteric fat is an independent determinant of fatty liver and that visceral fat is an important risk factor for cardiovascular disease rather than general obesity [17]. Furthermore, pro-inflammatory cytokines such as tumor necrosis factor alpha (TNFα), interleukin 1 beta (IL-1β), and monocyte chemoattractant protein-1 (MCP-1) as well as adipokine secretion (e.g., leptin and adiponectin), are affected by visceral fat hypertrophy [15,18].

In recently years, sugar consumption has markedly increased due to excessive intake of processed foods and beverages that contain large amounts of added sugar. Excessive sugar consumption leads to low-nutritional, high-density energy intake, and this can induce body weight gain and high levels of glucose, TG and cholesterol in circulation, as well as increased visceral fat deposits and liver fat accumulation [19,20]. The development of alternative sweeteners has the potential to reduce the adverse effects of sugar [19,20,21]. d-Xylose is a commercially available alternative sweetener and has been shown its beneficial effects on health. d-Xylose is a monosaccharide sugar that can act as a potential inhibitor of intestinal sucrase activity by preventing the conversion of sucrose into glucose and fructose [22]. Previous study has reported anti-obesity effects of d-xylose with reduced body weight, liver, subcutaneous and visceral fat. Improved lipid profiles and steatosis by regulating hepatic adipogenesis and lipogenesis genes [23]. Xylobiose (XB) is an alternative sweetener that is abundant in bamboo shoots and is composed of two xylose units that share a β-1,4 linkage. XB is also a major component of xylo-oligosaccharide (XOS) [24]. Once XB is consumed orally, it is not excreted from the body through urine or feces and it is not hydrolyzed by digestive enzymes that are present in saliva, gastric juices, and the intestine. Rather, XB is hydrolyzed by intestinal microbiota [25]. Administration of XOS has been reported to lower both blood glucose and serum lipid levels [26,27]. In addition, XOS has been shown to increase the production of short chain fatty acids in the colon and to mediate bifidogenic properties, thereby supporting a healthy environment in the gut [28,29]. XOS increased in bifidobacteria numbers and it has been shown to greatly affect *Lactobacillus* populations in both animals and humans [30,31]. Meanwhile, control of hepatic lipogenesis has been reported to be an anti-diabetic effect of XB [32]. Although numerous studies regarding effect of d-xylose and XOS on metabolic dysregulation have reported, there was lack of reported evidence about XB. Based on these observations, the goal of the present study was to investigate whether XB regulates obesity and related adipogenic metabolic dysfunction.

## 2. Results

### 2.1. Effects of XB on Body Weight and Various Adipose Tissue Weights

**Figure 1:**
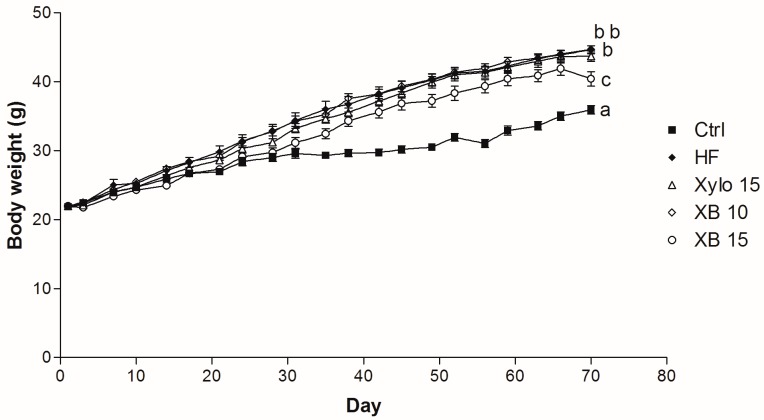
Effect of XB on body weight (b.w.) changes. b.w. of the five experimental groups are presented longitudinally. Ctrl, control mice that received the AIN93G diet; HF, obesity control mice that received a HFD; Xylo 15, mice that received a HFD with 15% of the total sucrose replaced with d-xylose; XB 10, mice that received a HFD with 10% of the total sucrose replaced with xylobiose; XB 15, mice that received a HFD with 15% of the total sucrose replaced with xylobiose.

High fat diet (HFD) administration for ten weeks induced obesity in C57BL/6 mice (Table 1). Animals that received a HFD for ten weeks had body weights that were significantly and consistently higher compared to the Ctrl group from week 3 (*p* < 0.001) through week 10 (*p* < 0.001) (Figure 1). In contrast, the XB 15 group exhibited a significant reduction in body weight compared to the HF group (*p* < 0.001). Food intakes were not different among the groups (Table 1). Weights of the mesenteric, subcutaneous, and perirenal adipose tissues were also decreased in XB supplementation compared to the HF group. Moreover, total adipose tissue weight for the XB 15 group was significantly lower compared to the HF group (*p* < 0.01).

### 2.2. Effects of XB on Blood Profiles

Blood profiles, including fasting blood glucose, plasma lipid profiles, and levels of leptin and adiponectin were analyzed after ten weeks of supplementation (Table 2). Fasting blood glucose levels for the HF group exhibited a significant two-fold increase compared to the Ctrl group (*p* < 0.001). In contrast, the XB 15 group showed a 30% reduction in fasting blood glucose levels compared to the HF group (*p* < 0.001). Insulin levels in the HF group markedly increased compared to the Ctrl group (*p* < 0.001). In contrast, insulin levels in the d-xylose and XB supplementation groups significantly decreased by 39.37%, 43.45%, and 51.79%, respectively (*p* < 0.001 for all). The HOMA-IR values for the Xylo 15, XB 10, and XB 15 groups were reduced by 56%, 49%, and 67%, respectively, compared to the HF group (*p* < 0.001 for all). Taken together, these results indicate that both xylose and XB can contribute to anti-insulin resistance. TG and TC levels were also significantly higher in the HF group compared to the Ctrl group (*p* < 0.001), while these levels were decreased in the Xylo 15 (*p* < 0.01), XB 10 (*p* < 0.001), and XB 15 (*p* < 0.001) groups. Similarly, LDL-C and VLDL-levels were significantly lower in the Xylo 15 (*p* < 0.05), XB 10 (*p* < 0.001), and XB 15 (*p* < 0.001) groups compared to the HF group. Conversely, HDL-C levels tended to be lower in the HF group, yet were higher in the supplemented groups, although not significantly. The atherogenic index (AI) value for the HF groups was significantly higher compared with the Ctrl group (*p* < 0.001), while the AI values for the d-xylose and XB supplementation groups were lower (*p* < 0.001 for all).

Leptin hormone levels were elevated in the HF group (*p* < 0.001) and decreased in the XB 15 group (*p* < 0.01). In contrast, adiponectin hormone levels were significantly lower in the HF group (*p* < 0.001) compared to the Ctrl group, and these levels increased in the Xylo 15 (*p* < 0.001) and XB 15 (*p* < 0.01) groups compared to the HF group.

### 2.3. Effects of XB on Gene Expression Levels Related to Adipogenesis, Lipogenesis, and β-Oxidation in Mesenteric Adipose Tissues

**Figure 2:**
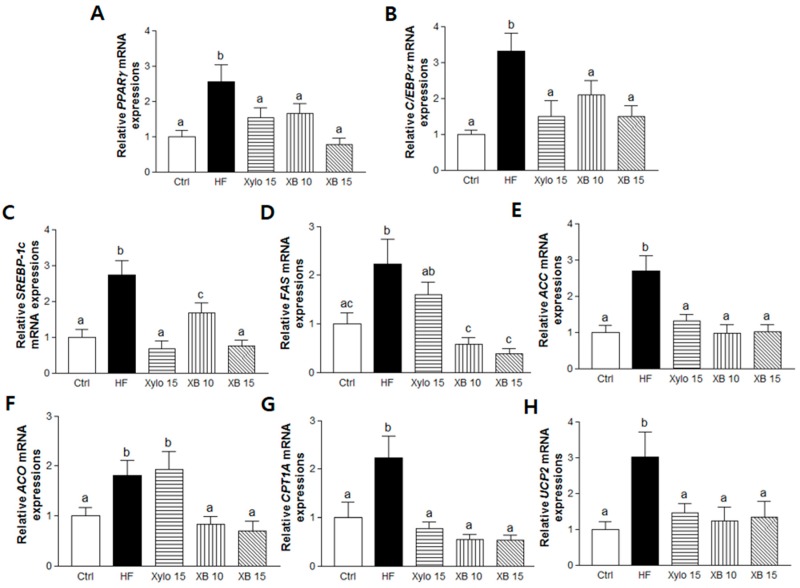
Effects of XB on the expressions of genes related to adipogenesis, lipogenesis, and energy metabolism in mesenteric adipose tissue. Real-time PCR was performed to detect mRNA levels of adipogenesis and lipogenesis-related genes in mesenteric adipose tissues. (**A**) *PPARγ*; (**B**) *C/EBPα*; (**C**) *SREBP-1c*; (**D**) *FAS*; (**E**) *ACC* and β-oxidation-related genes; (**F**) *ACO*; (**G**) *CPT1A*; and (**H**) *UCP2*. Detection of *GAPDH* was performed as a loading control. The values presented are the mean ± SEM and all of the data were analyzed with one-way ANOVA and Newman-Keuls’ post hoc test. The superscript letters indicate significant differences (*p* < 0.05). PPARγ, peroxisome proliferator-activated receptor gamma; C/EBPα, CCAAT/enhancer binding protein alpha, SREBP-1c, sterol regulatory element-binding protein 1; FAS, fatty acid synthase; ACC, acetyl-CoA carboxylase; ACO, acyl-CoA oxidase; CPT1A, carnitine palmitoyltransferase I A; UCP2, uncoupling protein 2; GAPDH, Glyceraldehyde 3-phosphate dehydrogenase; Ctrl, control mice that received the AIN93G diet; HF, obesity control mice that received a HFD; Xylo 15, mice that received a HFD with 15% of the total sucrose replaced with d-xylose; XB 10, mice that received a HFD with 10% of the total sucrose replaced with xylobiose; XB 15, mice that received a HFD with 15% of the total sucrose replaced with xylobiose.

Genes regulating adipogenesis, including, peroxisome proliferator-activated receptor gamma (*PPARγ*) and CCAT/enhancer-binding proteins alpha (*C/EBPα*), as well as lipogenic genes such as sterol regulatory element-binding protein 1c (*SREBP-1c*), fatty acid synthase (*FAS*), and acetyl-CoA carboxylase (*ACC*), were up-regulated in mesenteric adipose tissues from the HF group. Conversely, the mRNA levels of these genes were significantly down-regulated by d-xylose and XB supplementation (Figure 2A–E).

A HFD was also associated with up-regulation of β-oxidation-associated gene expressions, including that of acyl CoA oxidase (*ACO*), carnitine palmitoyltransferase I-A (*CPT1A)*, and energy metabolism-related genes, including mitochondrial uncoupling protein 2 (*UCP2*). In contrast, XB supplementation down-regulated these three genes, while d-xylose suppressed *CTP1A* and *UCP2* (Figure 2F–H).

### 2.4. Effects of XB on Fatty Acid Uptake, Lipolysis, and Related Pro-Inflammatory Cytokines in Mesenteric Adipose Tissues

**Figure 3:**
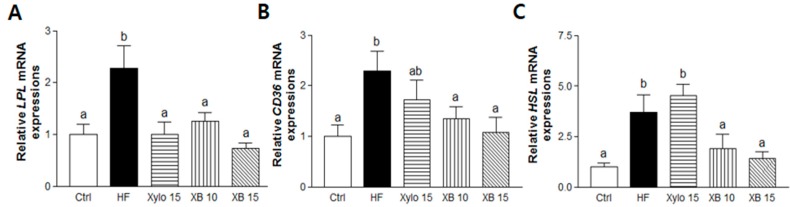

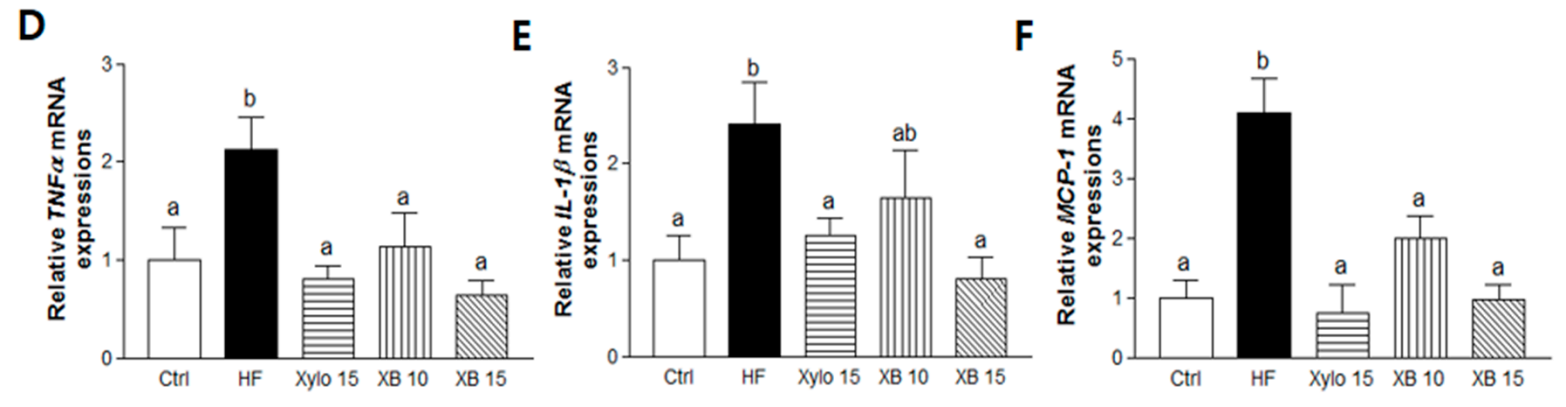
Effects of XB on the expressions of genes related to fatty acid uptake, lipolysis, and pro-inflammatory cytokines in mesenteric adipose tissues. Real-time PCR was performed to detect mRNA level of fatty acid uptake and lipolysis-related genes in mesenteric adipose tissues. (**A**) *LPL*; (**B**) *CD36*; and (**C**) *HSL*; (**D**) *TNFα*; (**E**) IL-1β; and (**F**) MCP-1. Detection of *GAPDH* was performed as a loading control. The values presented are the mean ± SEM and all of the data were analyzed with one-way ANOVA and Newman-Keuls’ post hoc test. The superscript letters indicate significant differences (*p* < 0.05). LPL, lipoprotein lipase; CD36, cluster of differentiation 36; HSL, hormone sensitive lipase; TNFα, tumor necrosis factor alpha; IL-1β, interleukin 1 beta; MCP-1, monocyte chemoattractant protein-1; Ctrl, control mice that received the AIN93G diet; HF, obesity control mice that received a HFD; Xylo 15, mice that received a HFD with 15% of the total sucrose replaced with D-xylose; XB 10, mice that received a HFD with 10% of the total sucrose replaced with xylobiose; XB 15, mice that received a HFD with 15% of the total sucrose replaced with xylobiose.

Lipoprotein lipase (*LPL*) and cluster of differentiation 36 (*CD36*) are fatty acid uptake markers in mesenteric adipose tissues and their levels were analyzed (Figure 3A,B). Compared to the HF group, XB supplementation significantly reduced fatty acid uptake potential by down-regulating *LPL* expression (*p* < 0.01 for XB 10, *p* < 0.001 for XB 15). Similarly, XB supplementation significantly reduced adipocyte membrane fatty acid uptake by regulating *CD36* expressions (*p* < 0.05 for both XB 10 and XB 15). The mRNA level of a key lipolysis regulating gene, hormone-sensitive lipase (*HSL*), was also up-regulated in the HF group. However, this increase was suppressed by XB supplementation (*p* < 0.05 for XB 10, *p* < 0.01 for XB 15) (Figure 3C).

Pro-inflammatory cytokines are secreted by adipocytes and they contribute to chronic inflammation in obesity [33]. Therefore, we next investigate the expressions of pro-inflammatory cytokines, including *TNFα*, *IL-1β*, and *MCP-1*. The mRNA levels of all three pro-inflammatory cytokines were significantly higher in the HF group compared to the Ctrl group, while all three levels were lower in the D-xylose and XB supplementation groups (Figure 3D–F).

### 2.5. Effects of XB on Lipid Accumulation and Lipogenesis in the Liver

**Figure 4:**
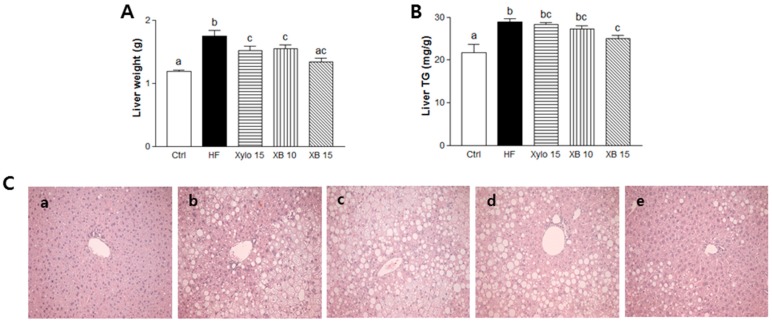

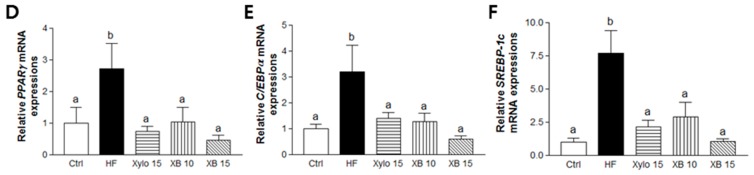
Effects of XB on lipid accumulation and lipogenesis in the liver. (**A**) Liver weight (g); (**B**) Liver triglyceride content (mg/g). (**C**) Hepatic histopathologic features (**a**) Ctrl; (**b**) HF; (**c**) Xylo 15; (**d**) XB 10; and (**e**) XB 15. Magnification ×200; (**D**–**F**) Real-time PCR results of adipogenesis and lipogenesis-related genes expression in the liver. The values presented are the mean ± SEM and all of the data were analyzed with one-way ANOVA and Newman-Keuls’ post hoc test. The superscript letters indicate significant differences (*p* < 0.05). *PPARγ*, peroxisome proliferator-activated receptor gamma; *C/EBPα*, CCAAT/enhancer binding protein alpha, *SREBP-1c*, sterol regulatory element-binding protein 1; Ctrl, control mice that received the AIN93G diet; HF, obesity control mice that received a HFD; Xylo 15, mice that received a HFD with 15% of the total sucrose replaced with d-xylose; XB 10, mice that received a HFD with 10% of the total sucrose replaced with xylobiose; XB 15, mice that received a HFD with 15% of the total sucrose replaced with xylobiose.

The mice that received a HFD exhibited significant increase in liver weight compared to the Ctrl group (Figure 4A *p* < 0.001). Conversely, both d-xylose and XB supplementation inhibited liver weight gain. In particular, liver weight in the XB 15 group almost recovered to the level of Ctrl group. In addition, hepatic TG accumulation decreased by 15% with XB supplementation (Figure 4B *p* < 0.05) and fewer large fat droplets were observed with d-xylose and XB supplementation (Figure 4C). The mRNA level of genes associated with lipogenesis, including *PPARγ*, *C/EBPα*, and *SREBP-1c*, were significantly increased in the HF group compared to the Ctrl group (Figure 4D–F; *p* < 0.05 for *PPARγ* and *C/EBPα*; *p* < 0.001 for *SREBP-1c*). Meanwhile, both d-xylose and XB supplementation down-regulated the expression levels of these genes compared to the HF group.

## 3. Discussion

The results of the present study demonstrate that XB, an alternative sugar, exhibits anti-obesity effects by regulating obesity-associated metabolic alterations, fat accumulation, and inflammation. Specifically, XB suppressed body weight gain, and weight gain in several types of adipose tissue, while also inducing a significant beneficial effect in regulating plasma lipid profiles and AI in HFD-induced obesity. Correspondingly, molecular analyses of the mesenteric tissues showed that XB supplementation down-regulated genes associated with lipogenesis, adipogenesis, lipid uptake, lipolysis, and related pro-inflammatory cytokines. In addition, XB supplementation protected HFD-induced hepatic lipid accumulation by regulating related hepatic lipogenesis genes expressions.

Added sugar in foods contributes to high-density energy intake and a positive energy balance. The latter is a major risk factor for obesity due to its ability to cause hypertrophy and hyperplasia of adipocytes as a result of excessive energy and nutrients being stored [20,34]. However, energy balance cannot be precisely predicted based on the amount of food that is consumed since calorie absorption in the gut, basal energy expenditure, and functional responses of organs to utilize nutrients for energy can vary due to various factors, including hormones and neuronal factors, as well as psychological and environmental backgrounds [35]. Diets high in sucrose and fat have been shown to induce leptin resistance, escalated serum TG content, and down-regulate adiponectin levels. Moreover, these alterations were reversed with a sugar-free diet [36].

XB is a major component of XOS and has 30% the sweetness of sucrose [37]. Studies on the health effects of XOS have suggested that it lowers TG, cholesterol, and glucose levels in blood [26,27]. XOS is considered to be a prebiotic since it helps to generate short chain fatty acids in the colon and it improves microbiota balance in the intestine [28,38]. Moreover, XOS is associated with a decreased risk of inflammatory responses, T2DM, and opportunistic pathogens induced damage [27]. XB is similar to XOS in its potential to be used in food to improve energy balance and metabolic efficiency [37]. Correspondingly, improved diabetes-related metabolic changes have been observed in db/db mice supplemented with XB, particularly in relation to control of hepatic lipogenesis [32].

The observed increases in body weight and changes in plasma lipid profiles in the present study are consistent with changes previously shown to be associated with obesity and related diseases, including NAFLD and dyslipidemia. XB supplementation decreased body weight and body fat in the obese mice and significantly suppressed plasma levels of TGs and cholesterols. AI values highly correlate with atherogenic profiles, namely apoB containing lipoproteins and smaller LDL particles. While plasma TGs and HDL-C independently represent coronary risk factors, the AI formula that uses both these factors (e.g., log(TG/HDL-C)) is more precise and useful in predicting plasma atherogenicity [39,40]. AI values were significantly higher in the HF group, and this increase was suppressed with XB supplementation. These results indicate that XB may have a beneficial effect on the metabolic syndrome characteristics induced by a HFD that are defined by ATP III, 2001 [41].

XB supplementation was also found to regulate plasma levels of leptin and adiponectin. Adipocytes mediate endocrine functions via secretion of leptin and adiponectin. Leptin controls satiety signals and regulates food intake for energy homeostasis [42]. However, reduced sensitivity to leptin despite elevated levels of this hormone is often associated with abdominal obesity [43,44]. Peripheral leptin targets the liver, adipocytes, and blood, and its functions include regulating nutrition absorption, insulin sensitivity, inhibition of sugar uptake, and suppression of lipogenesis in WAT [45,46,47]. Meanwhile, adiponectin is a 30 k Da adipokine that is produced in adipose tissues and its levels inversely correlate with obesity to regulate glucose and lipid metabolism, as well as insulin sensitivity [48,49]. In the present study, XB supplementation significantly increased levels of both leptin and adiponectin, while reduced fasting blood glucose and liver TGs. Taken together, these results suggest that XB may have benefits for obesity-related metabolic dysregulation.

XB supplementation was found to significantly suppress fat accumulation in mesenteric (29%), subcutaneous (22%), and perirenal (16%) deposits. To further investigate the possible molecular mechanism(s) responsible for regulating mesenteric fat accumulation, both adipogenic and lipogenic gene expressions were analyzed. *PPARγ* is a key regulating gene in adipogenesis, in coordination with C/EBPα a, based on its ability to stimulate preadipocyte differentiation and the generation of mature adipocytes and to enhance lipid accumulation [35,50]. *SREBP-1c* is a transcription factor that contributes to the biosynthesis of fatty acids and TG and it is highly expressed in the liver and in WAT. SREBPs activate FAS and ACC, which promote the production of acetyl-CoA and NADPH for lipogenesis [51]. In the present study, XB supplementation inhibited expression of the adipogenic genes, *PPARγ* and *C/EBPα* and expression of the lipogenic genes, *SREBP-1c*, *FAS,* and *ACC*. These changes in gene expression may have contributed to the observed reduction in fat tissue weights, including that of mesenteric fat.

It has been proposed that adipocytes regulate energy balance. Furthermore, excess lipid intake over a long period of time has been shown to affect fat metabolism and to induce a re-equilibration of lipid balance due to its effect on fat oxidation [35,52]. Increased body fat has also been associated with an increase in fat oxidation, and this favors long-term regulation of lipid balance and body weight stability, thereby representing a metabolic adaptation caused by excess fat intake [35]. CPT1A initiates β-oxidation by shuttling FFAs to the mitochondrial matrix [53]. Upon reaching the matrix, ACO is the first enzyme to act on these substrates during the process of β-oxidation [54]. In the present study, expression levels of *CPT1A* and *ACO* were elevated in the HF group and decreased in the groups receiving XB supplementation. It can be suggested that decreased amount of adipocyte accounts for decreased β-oxidation processing for XB groups. UCP2 is expressed in WAT and it is able to partially uncouple respiration in mitochondria, thereby leading to reduced efficiency of β-oxidation. *UCP2* was increased by HFD in response to enhanced β-oxidation as resistance to obesity or defense mechanism [35,55,56]. In the present study, mRNA levels of *UCP2* were up-regulated concomitant with β-oxidation in mesenteric adipocytes in the HFD groups and down-regulated by XB supplementation. Thus, it is possible that a decrease in adipocyte mass due to XB supplementation could reduce an overcompensatory effect and metabolic fat changes that are induced by intake of HFD intake and this would help recover them to levels of the Ctrl group. Further studies are need to better understand the capacity for XB to regulate energy metabolism.

Obesity induces low-grade chronic systematic inflammation and the production of pro-inflammatory cytokines [57]. For example, macrophages in the adipocytes produce and secrete excessive proinflammatory cytokines and other leukocytes subsets, including master cells and T-cells in adipocytes also contribute to regulate inflammation [58]. The adipose tissues are a significant source of TNFα, IL-6, and resistin, and these cytokines are stimulated by an infusion of lipids or a HFD [12]. Serum concentrations of TNFα have been shown to be higher in obese individuals compared to non-obese controls [59] and among WAT, abdominal fat is more sensitive to inflammatory responses [60]. TNFα inhibits phosphorylation of the substrates of insulin receptors, and this leads to enhanced lipolysis of adipose tissues and the subsequent release of fatty acids into circulation [61]. In the present study, mRNA levels of *TNFα*, *IL-1β*, and *MCP-1* were up-regulated in mesenteric fat and lipolysis was enhanced by a HFD. In contrast, XB supplementation alleviated inflammation, lipolysis, and lipogenesis that was otherwise induced by a HFD diet.

CD36 and LPL are transporters of FFAs for cellular entry, and they also facilitate influxes of FFAs into adipocytes. As a result, adipocyte maturation is induced and fat mass increases. However, lipolysis in adipocytes is inhibited by insulin and suppressed by CD36 [62,63]. LPL is expressed in adipose tissue and then is released into plasma to catalyze the lipolysis of chylomicron and VLDL-C for adipocyte uptake by the cell surface receptor, CD36 [64]. The mice that received a HFD and became obese in the present study exhibited high levels of blood lipids and elevated levels of LPL and CD36 in adipocytes, which may be a response to metabolize blood lipids for storage. An increased influx of FFAs through CD36 from TG and VLDL lipolysis by LPL increased the NEFA pool in adipocytes for storage, thereby leading to larger adipocytes. HSL hydrolyzes TG that are stored in adipocytes and that contribute to plasma FFA levels by stimulating lipolysis. Up-regulation of HSL is closely associated with obesity and T2DM is associated with excessive lipids in plasma [65,66]. Lower levels of *LPL, CD36*, and *HSL* mRNA that were observed in the XB supplemented groups may indicate that reduced levels of blood lipid and fat mass to store FFA by suppressing FA uptake and fat lipolysis through enzymatic and inflammatory actions. Consistently, previous study has reported that XB enhanced insulin sensitivity and decreased fat accumulation in the liver of db/db mice [32].

Mesenteric fat deposit is highly associated with steatosis caused by increased lipid influx into liver from adipose tissue together with increased de-novo lipid synthesis in liver [61,67]. Visceral fat lipolysis increased in obesity is important for increased FFA release into portal vein and subsequent NAFLD development [68]. Various adipose deposits in obesity are highly correlated with fatty liver pathology. In the present study, XB suppressed liver weight gain induced by HFD and liver TG accumulation by regulating hepatic lipogenesis genes, including *PPARγ*, *C/EBPα*, and *SREBP-1c*. These results suggest that reduced FFA by decreased lipolysis in adipocytes by XB supplementation may reduce the fat accumulation in the liver.

Previously, studies have shown d-xylose reduced blood glucose and insulin levels by inhibiting sucrase activity [22,69]. In the present study, d-xylose improved blood profiles and suppressed liver weight gain, and down-regulated adipogenesis, as well as inflammation-related genes in mesenteric adipose tissue. However, XB was more effective in regulating metabolic dysregulations in mesenteric adipose tissue than comparable dose of d-xylose. One possible difference between Xylo and XB may be the prebiotic effect of XB. It has been reported that a high fat diet changes the profile of microbiota population, and these changes can correlate with obesity-associated metabolic diseases [70]. Thus, prebiotics may modulate the composition and activity of microbiota that are affected by a high-fat diet. For example, *Bifidobacterium* supplement was shown to lower body and fat weights and to improve the level of blood cholesterol in high fat diet-induced obese Sprague-Dawley rats [71]. Prebiotic activity of XB has also been reported, and the number of *Bifidobacterium* strains and bacterial growth were higher with xylooligosaccharide supplementation than with xylose supplementation. Additional studies are needed to further investigate the effect of XB on microbiota in high-fat diet-induced obesity models.

There are several limitations in the present study. First, although TG and VLDL-C content are related with fatty acid content, direct measures of fatty acid alone were not conducted. Second, signaling pathways, including Akt/mTOR were not evaluated. Last, histological changes of adipocytes were not investigated due to inability of samples.

## 4. Materials and Methods

### 4.1. XB Prepration

XB was a kind gift from the TS Corporation R&D Center (Incheon, Korea). XB with nearly 98% purity was produced from XOS (XOS-95P, Shandong Loglive Bio-Technology Co., Ltd., Qingdao, China) by using simulated moving bed (SMB) chromatography. An Agilent 1100 series high-performance liquid chromatography (HPLC) system (Agilent, Santa Clara, CA, USA) equipped with a Sugar-Pak I column (Waters, Milford, MA, USA) and a universal refractive index detector was used to analyze XB [72]. Double-distilled water was used for the mobile phase. Purified XB was stored in an auto-desiccator (Sanpla Dry Keeper, Sanplatec Corp., Osaka, Japan).

### 4.2. Animals and Diet

C57BL/6 mice (5-weeks old) were purchased (Central Lab Animal Inc., Seoul, Korea) and then maintained in Plexiglass cages with free access to water with a 12 h light-dark cycle, 22 ± 2 °C temperature, and 50 ± 5% humidity.

After a 1-week acclimatization period to laboratory conditions, the mice were randomized into five groups: (a) a control group (Ctrl, *n* = 10) which received a diet of modified American Institute of Nutrition (AIN)-93G (17% of the calories are from fat; Unifaith Inc., Seoul, Korea); (b) a high fat diet (HFD)-induced obesity control group (HF, *n* = 13) which received a HFD comprised of 60% fat; (c) a xylose 15% group (Xylo 15) which received a HFD with 15% of the total sucrose replaced with d-xylose (*n* = 13); (d) an XB 10% group (XB 10, *n* = 13) which received a HFD with 10% of the total sucrose replaced by XB; and (e) an XB 15% group (XB 15, *n* = 12) which received a HFD with 15% of the total sucrose replaced by XB. Commercially available D-xylose was included as a positive control and was administered at a dose comparable to XB. The mice were maintained on these diets according to their group for ten weeks prior to sacrifice. Compositions of the various diets are listed in Table 3. Body weight and food intake were measured twice a week. This study was authorized by the Institutional Animal Care and Use Committee of Ewha Womans University (No: 16-035) and followed the guidelines.

### 4.3. Blood and Tissue Sample Preparation

After a 10-week experimental period, mice were subjected to fasting for 12 h before being euthanized with isoflurane (Piramal Critical Care, Bethlehem, PA, USA). Blood was collected from the inferior vena cava and stored in ethylenediamineteraacetic acid (EDTA) tubes. Plasma was separated at 4 °C with centrifugation (13,000× *g*) for 15 min. Livers and several adipose tissues (epididymal, subcutaneous, mesenteric, and perirenal fat) were dissected from each mouse and weighed. All of the samples were stored at −80 °C. In addition, sections of liver were stored in 10% formalin and portions of liver and mesenteric adipose tissues were embedded in RNAlater (Invitrogen, Carlsbad, CA, USA).

### 4.4. Biochemical Analysis of Blood Samples

Fasting blood glucose levels were measured from the tail vein just prior to sacrifice by using a portable glucometer (Roche, Mannheim, Germany). Insulin levels in the plasma were measured by ELISA (Crystal Chem, Downers Grove, IL, USA). Homeostasis model assessment-estimated insulin resistance (HOMA-IR) was calculated by (fasting plasma insulin (μg/L) × fasting blood glucose (mg/dL))/22.5 to estimate insulin resistance. Plasma concentrations of TG, total cholesterol (TC), and high-density lipoprotein cholesterol (HDL-C) were analyzed with a commercially available kit (Asan Pharmaceutical, Seoul, Korea). Plasma leptin and adiponectin were quantified with enzyme-linked immunosorbent assay (ELISA) kits (Crystal Chem). Assays were performed according to the manufacturer’s instructions. Concentration of low-density lipoprotein cholesterol (LDL-C) and VLDL-C [73] as well as AI values [39,74], were calculated according to the following formulas. LDL-C = TC − HDL-C − (TG/5), VLDL-C = TG/5, and AI = log_10_ TG/HDL-C.

### 4.5. RNA Isolation and Quantitative Real-Time-PCR (RT-PCR)

Total RNA was isolated from mesenteric adipose tissues and liver tissues with TRIzol reagent (Invitrogen). Reverse transcription was performed by using RevertAid reverse transcriptase (Invitrogen) to generate cDNA. RT-PCR was subsequently performed with a Rotor-Gene^®^ Q system (Qiagen, Hilden, Germany) with 2X SYBR Green PCR master mix (Qiagen) as follows: 95 °C for 5 min, 95 °C for 15 s, and 60 °C for 10 s. The mRNA expression levels detected were normalized to *GAPDH* and relative mRNA levels were calculated according to the 2^−ΔΔCT^ method. The sequences of the primers used are listed in Table 4.

### 4.6. Histopathological Analysis and Liver TG Content Analysis

Left lateral liver lobes were isolated, immediately fixed in 10% phosphate buffered formalin and then embedded in paraffin wax. Paraffin sections (5 μm) were cut and deparaffinized in xylene for staining with hematoxylin and eosin. Stained liver sections were observed with an optical microscope (Olympus, Tokyo, Japan) by two independent pathologists. The liver sections were saponicated with ethanolic KOH solution (2:1 = 100% ethanol: 30% KOH) and TG content was analyzed with TG and free glycerol reagents (Sigma-Aldrich, St. Louis, MO, USA) as previously described [75].

### 4.7. Statistical Analyses

Data are presented as the mean ± standard error of the mean (SEM). The statistical significance was analyzed with GraphPad Prism software (GraphPad, San Diego, CA, USA) by using one way analysis of variance (ANOVA) followed by post hoc Newman-Keuls’ test. A probability level of *p* < 0.05 was considered statistically significant and data labeled with superscripted alphabet letters significantly differ.

## 5. Conclusions

**Figure 5:**
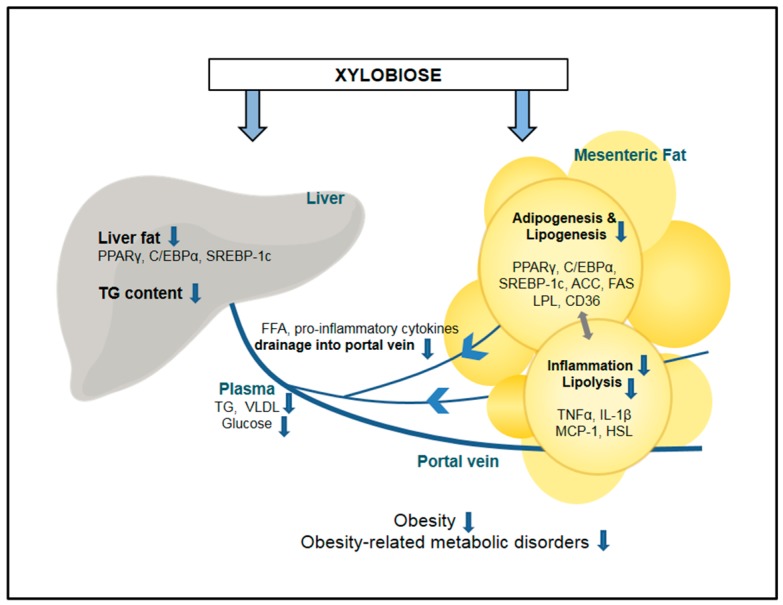
Proposed mechanism of xylobiose-regulated metabolic changes in obesity. Xylobiose suppresses adipogenesis, lipogenesis, lipolysis, and inflammation in mesenteric fat, and this reduces the release of FFAs and inflammatory cytokines to the portal vein. As a result, fat accumulation in the liver is suppressed. ACO, acyl-CoA oxidase; CD36, cluster of differentiation 36; C/EBPα, CCAAT/enhancer binding protein alpha; FAS, fatty acid synthase; FFA, free fatty acid; HSL, hormone-sensitive lipase; IL-1β, interleukin 1 beta; MCP-1, monocyte chemoattractant protein-1; LPL, lipoprotein lipase; PPARγ, peroxisome proliferator-activated receptor gamma; SREBP-1c, sterol regulatory element-binding protein 1; TG, triglyceride; TNFα, tumor necrosis factor alpha; VLDL, very low-density lipoprotein.

XB is an effective alternative sweetener that can alleviate health damages caused by obesity. XB reduced liver fat accumulation and mesenteric adipose tissue adipogenesis and lipogenesis, inflammatory responses, as well as alleviated blood lipid levels. Reduced adipogenesis and lipogenesis in mesenteric adipose tissues may lead to suppressed lipolysis and inflammatory responses. Consequently, blood lipid levels are lower due to reduced FFA release from adipocyte and cause the reduced hepatic TG accumulation and hepatic adipogenesis (Figure 5). While direct measurements are not analyzed in this study, alleviated fatty liver is associated with insulin resistance and glucose metabolism, which in turn affects blood markers and adipocytes. With the confirmation of anti-obesity effect of XB from this study, the extent of direct involvement in metabolism suggested should be further analyzed more specifically. Further in vivo and long-term clinical trials with various doses of XB supplementation needs to verify these effects and related signaling.

## Figures and Tables

**Table 1:** Body weight, food intake, and adipose tissue weights of mice fed with different diets for 10 weeks ^1^.

	Ctrl	HF	Xylo 15	XB 10	XB 15
Final body weight (g)	35.93 ± 0.60 ^a^	44.64 ± 0.58 ^b^	43.72 ± 0.75 ^b^	44.69 ± 0.51 ^b^	41.23 ± 0.76 ^c^
Food intake (g/day)Mesenteric adipose tissue (g)	3.32 ± 0.04	2.76 ± 0.03	2.87 ± 0.32	2.75 ± 0.03	2.89 ± 0.09
	0.56 ± 0.03 ^a^	1.4 ± 0.05 ^b^	1.3 ± 0.08 ^b^	1.38 ± 0.06 ^b^	0.99 ± 0.09 ^c^
Subcutaneous adipose tissue (g)	0.81 ± 0.11 ^a^	2.33 ± 0.11 ^b^	2.07 ± 0.12 ^bc^	1.91 ± 0.12 ^c^	1.81 ± 0.17 ^c^
Perirenal adipose tissue (g)	0.59 ± 0.04 ^a^	0.95 ± 0.02 ^b^	0.94 ± 0.04 ^b^	0.94 ± 0.03 ^b^	0.80 ± 0.04 ^c^
Epididymal adipose tissue (g)	1.31 ± 0.07 ^a^	1.85 ± 0.07 ^b^	2.04 ± 0.09 ^b^	1.93 ± 0.05 ^b^	1.91 ± 0.06 ^b^
Total adipose tissue (g)	3.27 ± 0.22 ^a^	6.53 ± 0.14 ^b^	6.35 ± 0.24 ^b^	6.15 ± 0.17 ^b^	5.52 ± 0.30 ^c^

^1^ The values presented are the mean ± SEM and all of the data were analyzed with one-way ANOVA and Newman-Keuls’ post hoc test. The superscript letters indicate significant differences (*p* < 0.05). Ctrl, control mice that received the AIN93G diet; HF, obesity control mice that received a HFD; Xylo 15, mice that received a HFD with 15% of the total sucrose replaced with d-xylose; XB 10, mice that received a HFD with 10% of the total sucrose replaced with xylobiose; XB 15, mice that received a HFD with 15% of the total sucrose replaced with xylobiose.

**Table 2:** Blood glucose, plasma lipid profiles, and atherogenic index of mice fed with different diets for 10 weeks ^1^.

	Ctrl	HF	Xylo 15	XB 10	XB 15
Fasting blood glucose (mg/dL)	75.00 ± 5.92 ^a^	188.46 ± 8.77 ^b^	146.77 ± 5.88 ^c^	171.62 ± 6.44 ^b^	130.75 ± 5.97 ^c^
Plasma insulin (μg/L)	0.44 ± 0.04 ^a^	3.24 ± 0.16 ^b^	1.97 ± 0.12 ^c^	1.83 ± 0.10 ^c^	1.56 ± 0.10 ^c^
HOMA-IR	1.50 ± 0.17 ^a^	27.00 ± 1.55 ^b^	12.79 ± 0.92 ^c^	14.00 ± 0.90 ^c^	8.97 ± 0.74 ^d^
Triglyceride (mg/dL)	150.61 ± 11.98 ^a^	204.6 ± 3.42 ^b^	123.55 ± 5.76 ^c^	119.7 ± 3.84 ^c^	119.67 ± 3.41 ^c^
Total cholesterol (mg/dL)	110.69 ± 3.97 ^a^	151.67 ± 6.54 ^b^	132.07 ± 4.94 ^c^	126.03 ± 3.34 ^ac^	112.56 ± 4.91 ^a^
LDL-cholesterol (mg/dL)	4.37 ± 4.01 ^a^	43.62 ± 7.46 ^b^	27.55 ± 4.49 ^c^	16.61 ± 2.56 ^a,c^	10.94 ± 3.1 ^a^
VLDL-cholesterol (mg/dL)	30.12 ± 2.4 ^a^	40.92 ± 0.69 ^b^	24.71 ± 1.15 ^c^	23.94 ± 0.77 ^c^	23.93 ± 0.68 ^c^
HDL-cholesterol (mg/dL)	76.2 ± 3.34 ^a,b^	67.13 ± 3.41 ^a^	79.81 ± 4.13 ^a,b^	85.48 ± 2.51 ^b^	77.69 ± 4.1 ^a,b^
Atherogenic index	0.29 ± 0.04 ^a^	0.49 ± 0.02 ^b^	0.19 ± 0.03 ^c^	0.15 ± 0.02 ^c^	0.19 ± 0.02 ^c^
Leptin (ng/mL)	14.44 ± 1.49 ^a^	30.85 ± 0.84 ^b^	29.35 ± 1.44 ^b,c^	29.42 ± 0.54 ^b,c^	25.54 ± 1.42 ^c^
Adiponectin (ng/mL)	1.11 ± 0.03 ^a^	0.84 ± 0.02 ^b^	0.98 ± 0.03 ^c^	0.88 ± 0.02 ^b^	0.96 ± 0.04 ^c^

^1^ The values presented are the mean ± SEM and all of the data were analyzed with one-way ANOVA and Newman-Keuls’ post hoc test. The superscript letters indicate significant differences (*p* < 0.05). Ctrl, control mice that received the AIN93G diet; HOMA-IR, Homeostasis model assessment-estimated insulin resistance; HF, obesity control mice that received a HFD; Xylo 15, mice that received a HFD with 15% of the total sucrose replaced with d-xylose; XB 10, mice that received a HFD with 10% of the total sucrose replaced with xylobiose; XB 15, mice that received a HFD with 15% of the total sucrose replaced with xylobiose.

**Table 3:** Compositions of the diets used in the experiment.

Ingredient	AIN-93G ^1^	High Fat ^2^	Xylo 15 ^3^	XB 10 ^4^	XB 15 ^5^
	(g)	(g)	(g)	(g)	(g)
Casein, lactic	200	200	200	200	200
l-Cystine	3	3	3	3	3
Corn starch	397.5	0	0	0	0
Maltodextrin	132	116.4	116.4	116.4	116.4
Sucrose	100	77.4	65.8	69.6	65.8
d-Xylose	0	0	11.6	0	0
Xylobiose	0	0	0	7.7	11.6
Cellulose	50	50	50	50	50
Soybean oil	70	25	25	25	25
Lard	-	245	245	245	245
Mineral mix	35	10	10	10	10
Dicalcium phosphate	-	13	13	13	13
Calcium carbonate	-	5.5	5.5	5.5	5.5
Potassium citrate·H_2_O	-	16.5	16.5	16.5	16.5
Vitamin mix	10	10	10	10	10
Choline bitartrate	2.5	2	2	2	2
Total amount	1000	773.85	773.85	773.75	773.85
Energy (kcal/g)	4	5.24	5.24	5.24	5.24

^1^ AIN-93G diet fed to Ctrl group; ^2^ High fat diet fed to HF group; ^3^ Sucrose was replaced with xylose at 15% (Xylo15) total amount of sucrose; ^4^ Sucrose was replaced with xylobiose at 10% (XB 10) total amount of sucrose; ^5^ Sucrose was replaced with xylobiose at 15% (XB 15) total amount of sucrose.

**Table 4:** Primer sequences used in real-time PCR.

Genes	Gene Symbol	GenBank ID	Forward Primer (5′ to 3′)	Reverse Primer (5′ to 3′)
*ACC*	Acaca	107476	AGGATTTGCTGTTTCTCAGAGCTT	CAGGATCTACCCAGGCCACAT
*ACO*	Acox1	11430	TTGGAAACCACTGCCACATA	AGGCATGTAACCCGTAGCAC
*CD36*	Cd36	12491	GTGCTCTCCCTTGATTCTGC	TGAGAATGCCTCCAAACACA
*C/EBPα*	Cebpa	12606	CCAAGAAGTCGGTGGACAAGA	CGGTCATTGTCACTGGTCAACT
*CPT1A*	Cpt1a	12894	AACCCAGTGCCTTAACGATG	GAACTGGTGGCCAATGAGAT
*FAS*	Fasn	14104	TGTGAGTGGTTCAGAGGCAT	TTCTGTAGTGCCAGCAAGCT
*HSL*	Lipe	16890	GGGCTGTCAAGCACTGT	GTAACTGGGTAGGCTGCCAT
*IL-1* *β*	Il1b	16176	ATGGCAACTGTTCCTGAACTCAACT	CAGGACAGGTATAGATTCTTTCCTTT
*MCP-1*	Mcpt1	17224	CCCACTCACCTGCTGCTACT	TCTGGACCCATTCCTTCTTG
*LPL*	Lpl	16956	TCCCGTTACCGTCCATCC	GAGTTTGACCGCCTTCCG
*PPARα*	Ppara	19013	CTCTGGGCAAGAGAATCCAC	ACTGGCAGCAGTGGAAGAAT
*PPARγ*	Pparg	19016	GAGCACTTCACAAGAAATTACC	GAACTCCATAGTGGAAGCCT
*SREBP-1c*	Srebf1	20787	TAGAGCATATCCCCCAGGTG	GGTACGGGCCACAAGAAGTA
*TNFα*	Tnfaip1	21926	TACGCCACAGAGAAGAAGCA	TGGCCTCCAGTAAGGAATTG
*UCP2*	Ucp2	22228	CACTTTCCCTCTGGATACCG	GTGCTCTGGTATCTCCGACC
*GAPDH*	Gapdh	14433	AACTTTGGCATTGTGGAAGG	TGTGAGGGAGATGCTCAGTG

*ACC*, acetyl-CoA carboxylase; *ACO*, acyl-CoA oxidase; *CD36*, cluster of differentiation 36; *C/EBPα*, CCAAT/enhancer binding protein alpha; *CPT1A*, carnitine palmitoyltransferase I A; *FAS*, fatty acid synthase; *HSL*, hormone-sensitive lipase; *IL-1β*, interleukin 1 beta; *MCP-1*, monocyte chemoattractant protein-1; *LPL*, lipoprotein lipase; *PPARα*, peroxisome proliferator-activated receptor alpha; *PPARγ*, peroxisome proliferator-activated receptor gamma; *SREBP-1c*, sterol regulatory element-binding protein 1; *TNFα*, tumor necrosis factor alpha; *UCP2*, uncoupling protein 2; *GAPDH*, glyceraldehyde 3-phosphate dehydrogenase.
